# The potential of artificial intelligence to improve patient safety: a scoping review

**DOI:** 10.1038/s41746-021-00423-6

**Published:** 2021-03-19

**Authors:** David W. Bates, David Levine, Ania Syrowatka, Masha Kuznetsova, Kelly Jean Thomas Craig, Angela Rui, Gretchen Purcell Jackson, Kyu Rhee

**Affiliations:** 1 Division of General Internal Medicine, Brigham and Women’s Hospital, Boston, MA USA; 2 Harvard Medical School, Boston, MA USA; 3 Harvard T. H. Chan School of Public Health, Boston, MA USA; 4 Harvard Business School, Harvard University, Boston, MA USA; 5 IBM Watson Health, Cambridge, MA USA; 6 Department of Pediatric Surgery, Vanderbilt University Medical Center, Nashville, TN USA

**Keywords:** Health care, Scientific community

## Abstract

Artificial intelligence (AI) represents a valuable tool that could be used to improve the safety of care. Major adverse events in healthcare include: healthcare-associated infections, adverse drug events, venous thromboembolism, surgical complications, pressure ulcers, falls, decompensation, and diagnostic errors. The objective of this scoping review was to summarize the relevant literature and evaluate the potential of AI to improve patient safety in these eight harm domains. A structured search was used to query MEDLINE for relevant articles. The scoping review identified studies that described the application of AI for prediction, prevention, or early detection of adverse events in each of the harm domains. The AI literature was narratively synthesized for each domain, and findings were considered in the context of incidence, cost, and preventability to make projections about the likelihood of AI improving safety. Three-hundred and ninety-two studies were included in the scoping review. The literature provided numerous examples of how AI has been applied within each of the eight harm domains using various techniques. The most common novel data were collected using different types of sensing technologies: vital sign monitoring, wearables, pressure sensors, and computer vision. There are significant opportunities to leverage AI and novel data sources to reduce the frequency of harm across all domains. We expect AI to have the greatest impact in areas where current strategies are not effective, and integration and complex analysis of novel, unstructured data are necessary to make accurate predictions; this applies specifically to adverse drug events, decompensation, and diagnostic errors.

## Introduction

Adverse events related to unsafe care represent one of the top ten causes of death and disability worldwide, and a third to a half appear preventable^1^. Investments in reducing harm can lead to substantial savings, and more importantly improve patient outcomes.

Twenty years after the Institute of Medicine’s “To Err Is Human” report, problems with safety remain all too common^2^ despite patient-centered strategies to create a culture of safety; for example, implementation of inpatient checklists, and computerization of prescribing and bar-coding^3–6^. However, safety issues outside the hospital have received much less attention than hospital safety, yet care is increasingly being shifted outside the hospital.

The application of artificial intelligence (AI) has tremendous potential as a tool for improving safety, both inside and outside of the hospital, by providing solutions to predict harms, collect a variety of data including both new and already-available data, and as part of quality improvement initiatives. For instance, AI can provide decision support by identifying patients at high risk of hospital harm to guide prevention and early intervention strategies. Similarly, AI can be applied in outpatient, community, and home settings. When coupled with digital approaches, these technologies can improve communication between patients and healthcare providers to reduce the frequency of preventable harms. While existing data will be helpful, new data will be available through technologies like sensors which should improve predictions.

AI techniques, such as machine learning (ML), can be leveraged to provide clinical risk prediction to improve patient safety. Data-driven ML algorithms have advantages over rule-based approaches for risk prediction, as they allow simultaneous consideration of multiple data sources to identify predictors and outcomes. Healthcare organizations are increasingly implementing ML and other forms of AI to improve patient care and outcomes. However, substantial impacts to safety and reduction of associated costs related to safety issues will require further acceptance of these technologies across the larger ecosystem including regulatory agencies and the marketplace.

Evidence suggests that the majority of healthcare harms fall into the following domains: healthcare-associated infections (HAIs), adverse drug events (ADEs), venous thromboembolism (VTE), surgical complications, pressure ulcers, falls, insufficient decompensation detection, and diagnostic errors—including missed and delayed diagnoses^7,8^. These domains are centered around hospital harm, and other issues undoubtedly play a role, but these adverse events account for the bulk of harm in hospitals. The goal of this paper was to conduct a scoping review to evaluate if AI has the potential to improve healthcare safety by reducing the frequency of adverse events within these eight major domains of harm.

## Methods

This scoping review is reported in accordance with the Preferred Reporting Items for Systematic Reviews and Meta-Analyses extension for Scoping Reviews (PRISMA-ScR)^9^.

### Search strategy

A structured search was used to query MEDLINE (Ovid) for relevant articles published on or before October 25, 2019. Two main concepts of AI and patient safety, including the eight harm domains, were mapped to the most relevant controlled vocabulary using Medical Subject Headings (MeSH), and free-text terms were added where necessary. The full search strategy is provided in Supplementary Note 1.

### Inclusion and exclusion criteria

The scoping review included studies that focused on the application of AI for prediction, prevention, and/or early detection of events in each of the harm domains in hospital, outpatient, community, and home settings. No comparisons were required, and all study designs were considered for inclusion. Articles were excluded if they were not published in the English language or reported on the use of AI to measure the frequency of harm events (e.g., post-marketing surveillance of drugs). Applications in robotics were also excluded. Detailed inclusion and exclusion criteria are provided in Supplementary Table 1.

### Screening and data abstraction

Articles were screened in two stages using Covidence (Australia), a web-based review management tool. Titles and abstracts were screened for relevance, and eligible records were evaluated based on full-text articles by a single reviewer. Additional articles were identified through handsearching. For each article included in the scoping review, citation information was exported from Covidence into an Excel spreadsheet and harm domains were manually abstracted by a single reviewer.

### Scoping review

The characteristics of studies that reported on the use of AI to improve patient safety were summarized. The literature was narratively synthesized for each harm domain highlighting key examples of how AI can be leveraged for prediction, prevention, and/or early detection of patient harms. Selected examples of traditional and novel data sources that could be used to develop AI algorithms to improve patient safety were summarized in tabular form.

### Evaluation of the potential for AI to improve patient safety

The findings of the scoping review were considered in the context of incidence, cost, and preventability of events to evaluate the potential of AI for improving safety. Current literature reporting on incidence, cost, and preventability was summarized for the eight harm domains in tabular form. Cost estimates were adjusted to United States dollars (USD, 2019) using the Producer Price Index to facilitate comparisons across the domains^10^. Projections around the likelihood of AI to improve safety in each of the harm domains were made and attractive early targets were identified as part of the Discussion.

## Results

### Characteristics of included studies

**Fig. 1:**
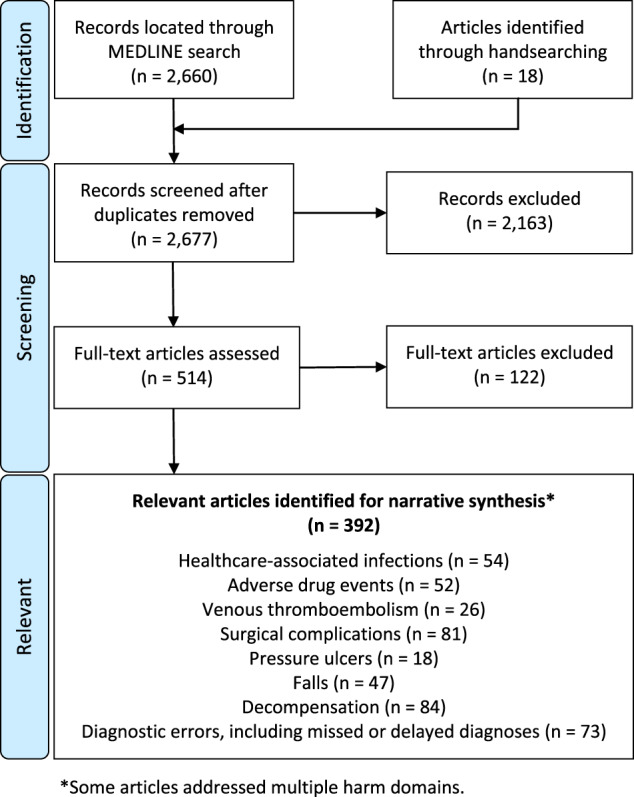
PRISMA flow diagram showing disposition of articles. The asterisk denotes that some studies addressed multiple harm domains.

From 2677 unique records, 392 articles met the inclusion criteria for the scoping review and are presented in Supplementary Table 2. A modified Preferred Reporting Items for Systematic Reviews and Meta-Analyses (PRISMA) flow diagram is provided in Fig. 1. The majority of studies were pre-clinical and relied on retrospective analyses of data. Most algorithms were not externally validated or tested prospectively. The incidence, cost, and preventability of events for each harm domain are presented in Table 1. Traditional and novel data sources that can be used to develop AI algorithms are presented in Table 2.

**Table 1:** Incidence, cost, and preventability of events in the eight harm domains from the peer-reviewed literature.

Patient safety domain	Population (years): incidence	Annual total cost adjusted to U.S. (2019) dollars^a^	Population: % preventable
Healthcare-associated infections	Inpatients (2015 data): 3.2%^11^	$10.7 billion^12^ [five significant HAIs]	Inpatients: 65% to 70%^13^ [CABSI or CAUTI]; 55%^13^ [VAP or SSI]
Adverse drug events	Inpatients (2014 data): 2.1%^22^ Present on admission (2014 data): 5.1%^22^	$30.0 billion^22^	Inpatients: 28%^23^
Venous thromboembolism	Inpatients (2013 review): 3.3%^7^	$15.1 to $30.4 billion^28^	Inpatient to 90 days after discharge: 70%^29^
Surgical complications	Inpatients (2014–2017 data): 16.0% within 30 days^34^ [invasive surgery]	$7.5 billion^35^ [emergency general surgery]	Inpatients: 42.1%^36^ [emergency non-trauma surgery]
Pressure ulcers	Inpatients (2009–2010 data): 2.7%^44^	$28.2 billion^45^	Inpatients: 97%^46^
Falls	Inpatients (2013 review): 1.1%^7^	$53.4 billion^51^	Inpatients: 87.5%^46^
Decompensation	Inpatients (2013 data): 3.6%^57^ [septicemia] Inpatients (2005–2015 data): 13.2%^58^ [failure-to-rescue after complications of trauma surgery]	$25.7 billion^57^ [septicemia]	Inpatients: 24.2%^58^ [failure-to-rescue after complications of trauma surgery]
Diagnostic errors	Outpatients (2014 review): at least 5.1%^72^	Exceeding $100 billion^b^ (ref. ^71^)	Unknown

*CABSI* catheter-associated bloodstream infection, *CAUTI* catheter-associated urinary tract infection, *HAI* healthcare-associated infection, *SSI* surgical site infection, *U.S.* United States, *VAP* ventilator-associated pneumonia.

^a^Estimates adjusted to U.S. (2019) dollars using the Producer Price Index^10^.

^b^Estimate not adjusted to U.S. (2019) dollars.

**Table 2:** Traditional and novel (italicized) data sources that can be used to develop artificial intelligence algorithms to improve patient safety; selected examples.

Patient safety domain	EHR	Claims	Risk scores	*Genome sequencing*	*Sensors*	*Computer vision*	*Other*
Healthcare-associated infections	X	X		*Of pathogens to monitor spread of nosocomial infections*	*Continuous vitals, Chemical vapor sensors*	*Of health care setting*	*Microbiology of random specimens, Smart sinks and dispensers*
Adverse drug events	X	X	BADRI model, Trivalle’s risk score	*Of patients for Cytochrome P450 polymorphisms*	*Continuous vitals, Continuous glucose monitoring*		*Patient report*
Venous thromboembolism	X	X	Padua prediction score, Khorana score	*Of patients for Cytochrome P450 polymorphisms, Factor V Leiden, Prothrombin 20210 mutations*	*Activity, Pressure, Location*		*Novel biochemical analytes*
Surgical complications	X	X	Surgical risk scores		*Continuous vitals*	*Of operating room*	
Pressure ulcers	X	X	Braden score		*Activity, Pressure, Location*	*Of bed*	*Novel biochemical analytes*
Falls	X	X	Hendrich model		*Activity, Pressure, Location*	*Of common spaces*	
Decompensation	X	X	MEWS, CART score		*Continuous vitals, Activity, Continuous glucose monitoring*		*Novel biochemical analytes*
Diagnostic errors	X	X			*Chemical vapor sensors*		*Clinician adjudication, Patient report*

Novel data sources italicized.

*BADRI* Brighton Adverse Drug Reactions Risk, *CART* Cardiac Arrest Risk Triage, *EHR* electronic health record, *MEW*, Modified Early Warning Score.

### Healthcare-associated infections

Approximately 3.2% of inpatients experienced HAIs in 2015 (ref. ^11^). The estimated annual cost for five significant HAIs is 10.7 billion (USD, 2019)^12^. Up to 70% of specific HAIs are considered preventable using existing evidence-based strategies^13^. The scoping review identified 54 articles (see Supplementary Table 2) describing the use of AI for prediction or early detection of HAIs.

ML and fuzzy logic (i.e., logical reasoning models based on incomplete or ambiguous data) have been applied for early detection of HAIs. Most algorithms were developed using claims-based data and information captured in electronic health records (EHRs) including laboratory test results and diagnostic imaging. With the integration of novel complex data, AI-based analytics could expedite detection and further improve diagnostic accuracy. For example, data from eNoses (i.e., chemical vapor sensors) have been analyzed using ML methods to rapidly detect ventilator-associated pneumonia (area under the curve (AUC) = 0.98), differentiate between six common wound pathogens (accuracy = 78%), and classify various strains of *Clostridium difficile* (sensitivities >80%; specificities >73%)^14–16^.

AI can also contribute to infection control by providing real-time, accurate predictions of HAI risk to guide patient-specific interventions before an infection occurs. For example, a random forest classification algorithm can predict onset of central line-associated bloodstream infections with an AUC of 0.82 (ref. ^17^).

AI can also play a role in improving adherence to existing safety protocols; for instance, computer vision using a convolutional network classifier has been applied to monitor hand hygiene compliance in the hospital setting (accuracy = 75%). Similarly, an ML algorithm was developed to provide real-time hand hygiene alerts in the outpatient setting based on data from multiple types of sensors, improving compliance from 54% to 100%^18,19^. These technologies are increasingly being applied to complex problems and could be used to improve other aspects of infection control, including sanitation or adherence to condition-specific safety protocols^20,21^.

### Adverse drug events

In 2014, ADEs were associated with 1.6 million hospitalizations in the U.S., totaling an estimated 30.0 billion (USD, 2019), with ~½ million ADEs occurring during hospital stays (2.1% of inpatients) and ~1 million present on admission (5.1% of admissions)^22^. About one in four ADEs are considered preventable given what is known today^23^. The review located 52 papers (see Supplementary Table 2) about leveraging AI to reduce the frequency of ADEs.

AI-based analytics can be applied to predict previously unreported ADEs based on drug similarities including chemical structure, mechanism of action, and polypharmacy side effects^24,25^. Deep learning methods using neural fingerprints have been shown to not only predict adverse drug reactions with an AUC of ~0.85, but also identify the associated molecular sub-structures^26^. These algorithms can inform the evidence-based development of safer medications. Similar techniques can be applied to predict drug–drug interactions for untested combinations of drugs^24^.

At the point of care, ML can be applied to analyze multiple datasets, including traditional patient data documented in EHRs (e.g., medical history, laboratory test results) with novel data (e.g., bioactivity of single nucleotide polymorphisms (SNPs)), to provide personalized ADE risk estimates and treatment recommendations to support decision making. Using genomic sequencing data, an artificial neural network (ANN) algorithm was developed to guide safer and more effective dosing of warfarin, predicting therapeutic dose with an accuracy of 83% in patients with international normalized ratios (INRs) >3.5 (ref. ^27^).

### Venous thromboembolism

Approximately 3.3% of inpatients develop VTEs, including deep venous thromboses (DVT) and pulmonary emboli (PE), with an estimated cost of 15.1–30.4 billion (USD, 2019) annually^7,28^. Adherence to current evidence-based strategies could reduce up to 70% of healthcare-associated VTEs^29^.

AI techniques can be used to identify patients at high risk for VTEs. The review located 26 articles (see Supplementary Table 2) about AI algorithms to prevent or safely rule out VTE. One study applied a super learner ensemble approach to identify inpatients at higher risk of future VTEs with an AUC of 0.69 (ref. ^30^). Prediction can also be applied to manage at-risk populations in the outpatient setting; for example, a multiple kernel learning algorithm was developed to predict VTE risk among patients undergoing chemotherapy with a sensitivity of 89%, markedly outperforming the recommended Khorana score (sensitivity = 11%)^31^.

AI methods could also recommend optimal patient-specific treatments. As described above, ML leveraging genomic sequencing data was used to guide safer warfarin dosing resulting in a reduced time to achieving a therapeutic INR (OR = 6.7) compared with standard clinical dosing^27^.

To date, AI has mostly contributed to VTE detection through the analysis of diagnostic imaging or radiologic reports. ML methods can also be applied to guide appropriate use of diagnostic imaging. For example, an ANN was applied to safely rule out DVT without ultrasonography in 38% of patients with a false-negative rate of only 0.2%^32^. Similarly, an ANN model was developed to guide computed tomography use for diagnosis of PE^33^. The algorithm achieved an AUC of 0.90 using an internal validation sample and 0.71 using external data, reiterating the importance of external validation for all AI or ML models.

### Surgical complications

Surgical complications are common; 16.0% of patients receiving invasive procedures experience a post-operative complication within 30 days^34^. Annual U.S. costs associated with complications following emergency general surgery are 7.5 billion (USD, 2019)^35^. It is estimated that 42.1% of complications following emergency non-trauma surgery are preventable^36^.

ML use cases include predicting adverse events in both the operative and post-operative setting. Eighty-one papers that leveraged AI to reduce surgical complications were located through the scoping review (see Supplementary Table 2). Predicting blood loss, need for prolonged post-operative intubation, post-operative mortality, pain, nausea, and vomiting all represent areas with demonstrated improvements to current risk tools^37–40^. For example, an ANN-based model achieved an accuracy of 92% at stratifying post-operative bleeding risk in patients undergoing cardiac pulmonary bypass^37^. Another ANN algorithm was developed to predict the need for prolonged ventilation after coronary bypass grafting (AUC = 0.71–0.73)^38^. Early intervention in these situations could translate into substantial improvements in patient safety.

An area of active research is the use of ML to recognize critical procedural steps in intra-operative videos. ANNs have been trained to identify the steps of laparoscopic sleeve gastrectomy procedures with an accuracy of 82%, and to determine whether the critical view of safety had been achieved in laparoscopic cholecystectomy videos, yielding an accuracy of 95%^41,42^. ML algorithms that can identify key operative components might be used in the future during procedures to warn surgeons of deviations from an expected sequence of steps or omission of critical elements. Other ML approaches in surgery on the horizon include computer precision pre-operative evaluation, augmented reality in the operating room, technical skills augmentation such as suturing, and ultimately autonomous robotic surgery^43^.

### Pressure ulcers

Approximately 2.7% of hospitalized patients in the U.S. develop a pressure ulcer^44^. The annual financial burden associated with treatment is estimated to be 28.2 billion (USD, 2019)^45^. Up to 97% of hospital-acquired pressure ulcers are preventable^46^.

The scoping review identified 18 articles (see Supplementary Table 2) that used AI for management of pressure ulcers. To date, most AI research in this area has focused on using sensor data for early detection; as such, using AI to predict future risk remains an area of opportunity. A recent study developed a random forest model, using EHR data to classify critical care patients based on their risk of developing pressure ulcers (AUC = 0.79 vs. 0.68 for the Braden Scale)^47^. Earlier studies tested the feasibility of using smart beds and wheelchair cushions for pressure ulcer detection using fuzzy logic and ML models, respectively^48,49^. Tracking data from embedded sensors, these algorithms detected a lack of movement and identified specific areas of skin that were at risk of developing an ulcer. Although the models were able to produce detection accuracy of up to 90% in experimental settings, their application and utility in notifying care providers and promoting early intervention remain uncertain.

### Falls

In 2014, 7.0 million fall-related injuries occurred among adults aged 65 and older^50^. These falls are estimated to account for 53.4 billion (USD, 2019)^51^. In the hospital, ~1.1% of inpatients experience a fall and 87.5% of these falls are considered preventable^7,46^. Forty-seven articles (see Supplementary Table 2) identified through the scoping review described the use of AI for prediction or early detection of falls.

AI approaches could be used to predict fall risk at the point of care using existing data from EHRs. For example, a support vector machine model was able to predict inpatient falls based on data documented from the previous day^52^. However, the model showed a sensitivity of 65% and a specificity of 70%, which are comparable to existing clinical risk assessments.

Many studies have applied ML methods for the early detection of falls. Classification models using data from wearable sensors in a laboratory setting showed relatively high levels of accuracy (54–84%) at stratifying subjects based on their risk of falls^53,54^. Using data from cameras, smart carpets, and wearable sensors intended for use in the home environment, support vector machine classifiers have been developed to detect falls, as well as to identify deviating gait patterns as predictors of future falls^55,56^. These models achieved accuracies of up to 100% in fall detection based on experimental and training datasets; however, their usability and applicability in real-world settings needs further testing.

### Decompensation

Clinical deterioration in the hospital remains common. For example, 3.6% of inpatients develop sepsis, costing an estimated 25.7 billion (USD, 2019) annually^57^. The failure-to-rescue rate following complications of trauma surgery, such as sepsis, is estimated at 13.2%, and one in four of these deaths are considered preventable^58^. However, prediction and early detection of decompensation remain a challenge in all areas of medicine.

The review located 84 papers (see Supplementary Table 2) that used AI to predict or detect the early signs of decompensation. Most research has focused on sepsis detection, which has seen improvements compared to traditional methods although, as with most ML algorithms, its generalizability may be poor^59–63^. It is likely that the detection of decompensation will improve by adding new categories of data, including biometric sensors such as continuous telemetry, motion activity sensors such as time spent in the bathroom or bedroom, novel biomarkers, and relevant patient-reported measures^64–69^. For example, ML has been used for early detection of sepsis using novel gene expression biomarkers with AUCs ranging from 0.86 to 0.92 (ref. ^68^). An AI tool has also been developed using a random forest model to predict nocturnal hypoglycemia from midnight to 6 am with an AUC of 0.84 based on continuous glucose monitoring to provide real-time feedback to inform optimal diabetes management before going to sleep^70^.

### Diagnostic errors

Diagnostic errors—both missed and delayed diagnoses—are relatively common in both inpatient and outpatient settings and estimated to occur in at least 5.1% of the U.S. population each year, with associated costs exceeding 100 billion (USD, 2016) annually^71,72^.

The scoping review identified 73 articles (see Supplementary Table 2) that leveraged AI to reduce diagnostic error. ML has widely demonstrated reduced errors in interpretation of imaging^73^. It has also proven beneficial for early diagnosis of lung cancer by analyzing exhaled breath using an eNose sensor; the support vector machine was able to classify cancer patients vs. non-cancer controls with a sensitivity of 87% and a specificity of 71%^74^. AI techniques are also being applied to reduce delays for critical diagnoses; for example, a clinical decision support system based on fuzzy logic was able to appropriately triage patients presenting to an emergency department with an accuracy of >99%—a 13% increase compared with traditional methods^75^.

A recent issue of the journal *Diagnostics* was devoted to this area^76^, and articles addressed diagnosis of a number of conditions. Another recent review summarized the main classes of problems that they believed AI systems are well suited to solve^77^.

## Discussion

Based on epidemiologic evidence and our scoping review, we believe that there are major opportunities to improve safety using data and AI across the eight domains to reduce the frequency of harm (Table 3). We expect AI to have the greatest impact in areas where current strategies are not effective, and integration and complex analysis of novel, unstructured data are necessary to make accurate predictions, which applies specifically to ADEs, decompensation, and diagnostic errors.

**Table 3:** Evaluation of the potential of artificial intelligence to improve patient safety in the eight harm domains.

Patient safety domain	Likelihood of impact
Healthcare-associated infections	AI may have a moderate impact on the reduction of HAIs given that current evidence-based practices are already effective when applied well.
Adverse drug events	AI can play a major role in ADE prevention. As more patients at risk of ADEs are accurately identified before a medication is administered or prescribed, a greater proportion of these events will become preventable. However, a key challenge lies in the lack of integrated high-quality datasets in which ADEs have been accurately captured. A variety of automated approaches have been effective at identifying patients likely to have experienced an ADE, but typically clinician adjudication is still required. ML could also help identify patients who may benefit from additional testing for specific single nucleotide polymorphisms to guide optimal drug therapy. These methods may also help to identify signals from the remainder of the genome beyond single nucleotide polymorphisms which may have prognostic impact.
Venous thromboembolism	We believe that AI will have a moderate effect on the reduction of VTE, as current evidence-based preventive strategies are already effective. AI solutions could provide further insights by identifying patients who could benefit from diagnostic testing for inherited thrombotic disorders to inform management of their condition.
Surgical complications	AI can be expected to have a moderate impact on the prediction and prevention of surgical complications both in the operating room and during recovery. Most complications felt to be preventable today are related to delayed diagnoses or intervention, technical issues, and infections. Given the overlap with other harm domains, focusing on advances in these other areas will likely also improve surgical safety.
Pressure ulcers	Pressure ulcers represent an attractive target with moderate to high potential for AI to prevent harm. Novel data sources such as motion and fluid sensors are now available, and large numbers of traditional clinical variables can be combined with the sensing data to predict who is at risk to guide evidence-based prevention.
Falls	AI is anticipated to have a moderate impact on fall prevention, given that this area has already received substantial attention and current risk mitigation strategies are effective. As with pressure ulcers, clinical data combined with sensing data can be used to predict when falls may occur, and which ones are likely to be associated with the most harm.
Decompensation	Leveraging novel data sources and AI has high potential to improve the prediction of decompensation to guide preventive strategies as well as early intervention to mitigate the impacts including premature death, given that current approaches are not effective. Given the serious nature of these events, preventing decompensation is a particularly attractive target. ML can deeply analyze data, beyond the standard values of heart rate or heart rate variability and will be critical to improving detection of decompensation and subsequent intervention.
Diagnostic errors	Diagnostic error is the most complex of the eight harm domains with vast opportunities for improvement using novel data sources and AI. ML could help to reduce the frequency of diagnostic errors by leveraging pattern recognition, bias minimization, and infinite capacity, areas where diagnosticians often falter. Although this area has garnered a lot of attention, many outstanding challenges remain, and current solutions only address a small fraction of what is possible. Most crucial to constructing valuable ML algorithms that help to reduce diagnostic error is the availability of large databases that accurately report errors.

*ADE* adverse drug event, *AI* artificial intelligence, *HAI* healthcare-associated infection, *ML* machine learning, *VTE* venous thromboembolism.

However, the application of AI and ML to improve patient safety is an emerging field and most of these algorithms have not yet been externally validated or tested prospectively. Promising performance based on development or internal validation samples may not translate into improvements in real-world practice. Algorithms may be limited in generalizability, and performance may be affected by the clinical context where the solution is implemented. Although the level of evidence is modest for all domains, we are highlighting what we believe to be the most promising areas.

Future research must focus on careful evaluation of clinical decision support systems based on AI analytics prior to widespread implementation to ensure safety and accuracy. From a technical perspective, candidate algorithms and tools should be validated at other sites, account for differential performance in subgroups, and explicitly report the uncertainty around any estimates or recommendations^78^. Furthermore, papers describing model development and performance assessments should adhere to reporting standards for transparency and provide important information about validity, biases, and generalizability to other settings^79^. Once high-quality AI solutions are developed, additional factors beyond performance must be considered to increase the likelihood of successful implementation and adoption by individual providers. There is an active area of research focused on identifying key barriers and facilitators to implementation of AI-based tools in healthcare^78,80,81^.

With data available today, especially laboratory information, imaging and continuous vital sign data, it should be possible to reduce the frequency of many types of harm. However, when the data are available, they are often unstructured, simply not in any documented form, or disputed. High-quality, large annotated databases will prove quite fruitful in minimizing patient harm in the future. New types of data, especially from the huge array of sensing technologies becoming available, but also including data from various other sources like information supplied directly by patients, genomic sequencing, and social media, offer new opportunities to improve predictions as the first step toward development of preventive interventions to improve safety. These types of data are becoming available and more accessible over time for research and to drive innovation^82–84^.

In addition, automated detection of safety issues of all types, but especially harm outside the hospital (e.g., post-marketing surveillance of drugs), will make routine measurement of the frequency of harm possible. While some of this will be rule-based, data-driven AI will also undoubtedly play a role.

This study has several limitations. The search query extracted evidence from a single database to identify published articles focused on the eight harm domains, and other literature may be available. Screening and data abstraction were completed by a single reviewer. The projections were informed by the incidence, cost, and preventability of harm as well as effectiveness of current strategies and promise of AI solutions.

## Conclusions

**Fig. 2:**
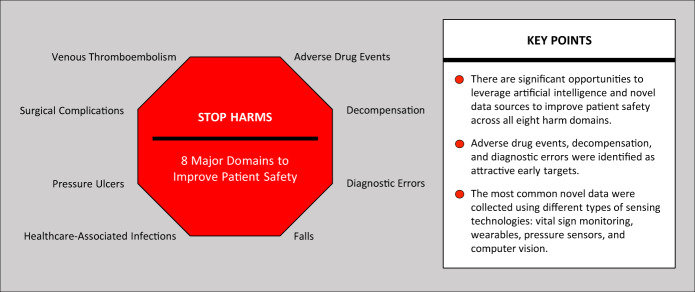
Summary of major domains of harm and key points. The first panel highlights the eight major domains of harm. The second panel summarizes the key points from the article.

Overall, AI has great potential to improve the safety of care (Fig. 2). In our view, harm domains including ADEs, decompensation, and diagnostic errors represent particularly attractive early targets. Transparent population-based datasets, which include diverse traditional (e.g., EHR, claims) and novel data (e.g., sensors, wearables, broader determinants of health), will be essential to build robust and equitable models. For AI to be effective, implementation of data-driven analytics will require organizations to develop, support, and iterate clinician, team, and system workflows for continued patient safety improvements.

## Supplementary information

Supplemental Information

## Data Availability

All data generated or analyzed during this study are included in this published article and its Supplementary Information.
