# Supplementary material for: The potential of artificial intelligence to improve patient safety: a scoping review
Source: NPJ Digit Med. 2021 Mar 19;4:54. doi: 10.1038/s41746-021-00423-6 (PMC7979747; doi:10.1038/s41746-021-00423-6)
Supplement: Supplementary file 1 — Supplemental Information [file 41746_2021_423_MOESM1_ESM.pdf]

SUPPLEMENTARY INFORMATION

Supplementary Note 1. MEDLINE (Ovid) search strategy (October 25, 2019)

1. exp Artificial Intelligence/ or ((Artificial or Comput* or Machine) adj1 Intelligence).ti,ab. or ((Machine or Deep) adj1 Learning).ti,ab. or Neural Network*.ti,ab. or Natural Language Processing*.ti,ab. or Computer Vision*.ti,ab.
2. exp Patient Safety/ or Patient Safet*.ti,ab.
3. (exp Patients/ or exp Inpatients/ or exp Hospitals/) and (exp Safety Management/ or exp Risk Management/ or exp Safety/)
4. exp Cross Infection/ or exp Infection Control/ or ((Nosocomial or Hospital or Cross or Health Care Associated or Healthcare Associated or Iatrogen*) adj1 Infection*).ti,ab.
5. exp "Drug-Related Side Effects and Adverse Reactions"/ or (Adverse Drug Event* or Adverse Drug Reaction* or Drug Side Effect* or Drug Toxicit*).ti,ab.
6. exp Venous Thrombosis/ or exp Pulmonary Embolism/ or (DVT or Deep Vein Thrombos* or Deep Venous Thrombos*).ti,ab. or (Pulmonary Emboli* or Pulmonary Thromboemboli*).ti,ab.
7. exp *Intraoperative Complications/ or exp *Postoperative Complications/ or ((Intraoperative or Peroperative or Postoperative or Surg*) adj1 Complication*).ti,ab. or Surgical injur*.ti,ab.
8. exp Accidental Falls/ or ((Inpatient or Patient) adj3 Fall*).ti,ab.
9. exp Pressure Ulcer/ or (Pressure ulcer* or Pressure sore* or Bedsore* or Bed sore*).ti,ab.

10. exp Clinical Deterioration/ or (Clinical Deterioration* or Decompensation*).ti,ab. or exp Sepsis/ or (Sepsis* or Septicemia* or Septic Shock*).ti,ab. or exp Failure to Rescue, Health Care/ or Failure to rescue*.ti,ab.
11. exp *Diagnostic Errors/ or exp *Delayed Diagnosis/ or ((Diagnos* adj3 (error* or delay* or miss*)) or Misdiagnos*).ti,ab.
12. 2 or 3 or 4 or 5 or 6 or 7 or 8 or 9 or 10 or 11
13. 1 and 12
14. limit 13 to (English language and humans)

Supplementary Table 1. Inclusion and exclusion criteria for the scoping review

Inclusion criteria	Exclusion criteria
Articles describing the application of AI for prediction, prevention, or early detection of events in eight harm domains:<br>- Healthcare-associated infections<br>- Adverse drug events<br>- Venous thromboembolism<br>- Surgical complications<br>- Falls<br>- Pressure ulcers<br>- Decompensation<br>- Diagnostic error	Articles focused on detection in general, rather than timely (i.e., early) detection (e.g., AI solutions to automate analysis of diagnostic imaging or radiologic reports)<br><br>Articles focused on the application of AI to measure the frequency of harm events (e.g., post-marketing surveillance of drugs, analysis of adverse event reporting systems)
Articles focused on inpatient, outpatient, community, and home settings	Articles describing applications in robotics
All article types (e.g., original research, reviews, and commentaries)	Articles not published in the English language

AI Artificial intelligence.

Supplementary Table 2. Data abstraction for studies included in the scoping review

Title of article	First author name	Year	PMID or citation	Harm domains
Evaluation of complications of kidney transplantation using artificial neural networks.	Abdolmaleki, P.	1997	9342099	Surgical complications
Big data cohort extraction for personalized statin treatment and machine learning.	Adam, T. J.	2019	30848466	Adverse drug events
Creation of an effective colorectal anastomotic leak early detection tool using an artificial neural network.	Adams, K.	2014	24337715	Surgical complications
High-accuracy detection of early Parkinson's Disease using multiple characteristics of finger movement while typing.	Adams, W. R.	2017	29190695	Diagnostic error
Artificial-intelligence-based hospital-acquired infection control.	Adlassnig, K. P.	2009	19745475	Healthcare-associated infections
Healthcare-associated infection surveillance and bedside alerts.	Adlassnig, K. P.	2014	24825687	Healthcare-associated infections
A fall prediction methodology for elderly based on a depth camera.	Alazrai, R.	2015	26737412	Falls
Predicting pressure injury in critical care patients: a machine-learning model.	Alderden, J.	2018	30385537	Decompensation
Passive in-home health and wellness monitoring: overview, value and examples.	Alwan, M.	2009	19964350	Falls
Impact of a Web-based diagnosis reminder system on errors of diagnosis.	Amy, L. R.	2006	17238463	Diagnostic error
Outcomes and complications after endovascular treatment of brain arteriovenous malformations: a prognostication attempt using artificial intelligence.	Asadi, H.	2016	27693769	Surgical complications
Feature selection for the accurate prediction of septic and cardiogenic shock ICU mortality in the acute phase.	Aushev, A.	2018	30457997	Decompensation
The use of privacy-protected computer vision to measure the quality of healthcare worker hand hygiene.	Awwad, S.	2019	29767747	Healthcare-associated infections
The effect of window size and lead time on pre-impact fall detection accuracy using support vector machine analysis of waist mounted inertial sensor data.	Aziz, O.	2014	25569889	Falls
A comparison of accuracy of fall detection algorithms (threshold-based vs. machine learning) using waist-mounted tri-axial accelerometer signals from a comprehensive set of falls and non-fall trials.	Aziz, O.	2017	27106749	Falls
A classification framework for exploiting sparse multi-variate temporal features with application to adverse drug event detection in medical records.	Bagattini, F.	2019	30630486	Adverse drug events
Development and performance of the pulmonary embolism result forecast model (PERFORM) for computed tomography clinical decision support.	Banerjee, I.	2019	31390040	Venous thromboembolism
Classifying bed inclination using pressure images.	Baran, P.	2014	25571032	Pressure ulcers
A comparative analysis of predictive models of morbidity in intensive care unit after cardiac surgery - part I: model planning.	Barbini, E.	2007	18034872	Decompensation; Surgical complications
Position recognition to support bedsores prevention.	Barsocchi, P	2013	23014763	Pressure ulcers
Enhanced neonatal surgical site infection prediction model utilizing statistically and clinically significant variables in combination with a machine learning algorithm.	Bartz-Kurycki, M. A.	2018	30078669	Healthcare-associated infections; Surgical complications
Postoperative vomiting in pediatric oncologic patients: prediction by a fuzzy logic model.	Bassanezi, B. S. B.	2013	22998327	Surgical complications
Integrated use of bedside lung ultrasound and echocardiography in acute respiratory failure: a prospective observational study in ICU.	Bataille, B.	2014	25144893	Diagnostic error
Detecting adverse events using information technology.	Bates, D. W.	2003	12595401	Adverse drug events; Falls; Healthcare-associated infections
New approach to risk determination: development of risk profile for new falls among community-dwelling older people by use of a Genetic Algorithm Neural Network (GANN).	Bath, P. A.	2000	10719768	Falls
A neural network aid for the early diagnosis of cardiac ischemia in patients presenting to the emergency department with chest pain.	Baxt, W. G.	2002	12447333	Diagnostic error
Falls risk prediction for older inpatients in acute care medical wards: is there an interest to combine an early nurse assessment and the artificial neural network analysis?.	Beauchet, O.	2018	29300432	Falls

Assessing patient risk of central line-associated bacteremia via machine learning.	Beeler, C.	2018	29661634	Healthcare-associated infections
A signal processing approach for detection of hemodynamic instability before decompensation.	Belle, A.	2016	26871715	Decompensation
AI tackles hospital infections: machine learning is helping clinicians.	Berglund, J.	2018	30452339	Healthcare-associated infections
Three different cases of exploiting decision support services for adverse drug event prevention.	Bernonille, S.	2011	21685623	Adverse drug events
Surgical risk is not linear: derivation and validation of a novel, user-friendly, and machine-learning-based predictive optimal trees in emergency surgery risk (POTTER) calculator.	Bertsimas, D.	2018	30124479	Healthcare-associated infections; Surgical complications
Predicting common maternal postpartum complications: leveraging health administrative data and machine learning.	Betts, K. S.	2019	30628159	Surgical complications
The potential for intelligent decision support systems to improve the quality and consistency of medication reviews.	Bindoff, I.	2012	22175237	Adverse drug events
Rapid, accurate, and on-site detection of C. difficile in stool samples.	Bomers, M. K.	2015	25823766	Diagnostic error
Neural network processing can provide means to catch errors that slip through human screening of pap smears.	Boon, M. E.	1993	8261846	Diagnostic error
Advanced technologies to curb healthcare-associated infections.	Boscart, V. M.	2009	19593077	Healthcare-associated infections
Who is a high-risk surgical patient?	Bose, S.	2018	30308542	Surgical complications
An automated detection system of drug-drug interactions from electronic patient records using big data analytics.	Bouzille, G.	2019	31437882	Adverse drug events
Continuous monitoring in an inpatient medical-surgical unit: a controlled clinical trial.	Brown, H.	2014	24342543	Decompensation
The HELP system and its application to infection control.	Burke, J. P.	1991	1679811	Healthcare-associated infections
Screening swabs surpass traditional risk factors as predictors of MRSA bacteremia.	Butler-Laporte, G.	2018	29890954	Healthcare-associated infections
Full-text automated detection of surgical site infections secondary to neurosurgery in Rennes, France.	Campillo-Gimenez, B.	2013	23920620	Healthcare-associated infections; Surgical complications
A comparative analysis of predictive models of morbidity in intensive care unit after cardiac surgery - part II: an illustrative example.	Cevenini, G.	2007	18034873	Decompensation; Surgical complications
Predicting hospital-acquired infections by scoring system with simple parameters.	Chang, Y. J.	2011	21887234	Healthcare-associated infections
Detecting evidence of intra-abdominal surgical site infections from radiology reports using natural language processing.	Chapman, A. B.	2017	29854116	Surgical complications
Detecting adverse drug events with rapidly trained classification models.	Chapman, A. B.	2019	30649737	Adverse drug events
Prediction of feature genes in trauma patients with the TNF rs1800629 A allele using support vector machine.	Chen, G.	2015	26117649	Decompensation
Artificial neural network: a method for prediction of surgery-related pressure injury in cardiovascular surgical patients.	Chen, H. L.	2018	29189496	Surgical complications
Use of an artificial neural network to predict risk factors of nosocomial infection in lung cancer patients.	Chen, J.	2014	25041000	Healthcare-associated infections
Use of an artificial neural network to construct a model of predicting deep fungal infection in lung cancer patients.	Chen, J.	2015	26163648	Healthcare-associated infections
The application of artificial neural networks and decision tree model in predicting post-operative complication for gastric cancer patients.	Chien, C. W.	2008	18705347	Surgical complications
Heart rate variability based machine learning models for risk prediction of suspected sepsis patients in the emergency department.	Chiew, C. J.	2019	30732136	Decompensation
Responses of staff nurses to an emr-based clinical decision support service for predicting inpatient fall risk.	Cho, I.	2019	31438275	Falls
Analytical performance of Envisia: a genomic classifier for usual interstitial pneumonia.	Choi, Y.	2017	29149880	Diagnostic error
Multicenter comparison of machine learning methods and conventional regression for predicting clinical deterioration on the wards.	Churpek, M. M.	2016	26771782	Decompensation
Artificial neural networks as prediction tools in the critically ill.	Clermont, G.	2005	15774070	Decompensation
Sepsis reconsidered: Identifying novel metrics for behavioral landscape characterization with a high-performance computing implementation of an agent-based model.	Cockrell, C.	2017	28728997	Decompensation
Use of advanced machine-learning techniques for noninvasive monitoring of hemorrhage.	Convertino, V. A.	2011	21795890	Decompensation

Development and validation of machine learning models to identify high-risk surgical patients using automatically curated electronic health record data (Pythia): A retrospective, single-site study.	Corey, K. M.	2018	30481172	Decompensation; Surgical complications
Semantic processing to identify adverse drug event information from black box warnings.	Culbertson, A.	2014	25954348	Adverse drug events
MRI features predictive of negative surgical margins in patients with HER2 overexpressing breast cancer undergoing breast conservation.	Dashevsky, B. Z.	2018	29321645	Diagnostic error
Assessing the clinical uses of fuzzy detection results in the automated detection of CVC-related infections: a preliminary report.	de Bruin, J. S.	2012	22874257	Healthcare-associated infections
Detecting borderline infection in an automated monitoring system for healthcare-associated infection using fuzzy logic.	de Bruin, J. S.	2016	27156053	Healthcare-associated infections
Decision support system for triage management: A hybrid approach using rule-based reasoning and fuzzy logic.	Dehghani, S.	2018	29673601	Diagnostic error
Design, implementation and evaluation of a clinical decision support system to prevent adverse drug events.	Del Fiol, G.	2000	11187651	Adverse drug events
Comparison of two knowledge bases on the detection of drug-drug interactions.	Del, F.	2000	11079867	Adverse drug events
Towards precision medicine: accurate predictive modeling of infectious complications in combat casualties.	Dente, C. J.	2017	28538622	Healthcare-associated infections
A decision model to predict the risk of the first fall onset.	Deschamps, T.	2016	27114199	Falls
Predicting adverse drug reactions through interpretable deep learning framework.	Dey, S.	2018	30591036	Adverse drug events
Artificial intelligence in clinical and genomic diagnostics.	Dias, R.	2019	31744524	Diagnostic error
ADESSA: a real-time decision support service for delivery of semantically coded adverse drug event data.	Duke, J. D.	2010	21346964	Adverse drug events
Predictive modeling for blood transfusion after adult spinal deformity surgery: a tree-based machine learning approach.	Durand, W. M.	2018	29215501	Surgical complications
Learning a severity score for sepsis: a novel approach based on clinical comparisons.	Dyagilev, K.	2015	26958288	Decompensation
Reduction of false arrhythmia alarms using signal selection and machine learning.	Eerikainen, L. M.	2016	27454128	Decompensation
Designing a social and assistive robot for seniors.	Eftring, H.	2016	27220732	Falls
Predicting active pulmonary tuberculosis using an artificial neural network.	El-Solh, A. A.	1999	10531161	Healthcare-associated infections
Predicting the presence of acute pulmonary embolism: a comparative analysis of the artificial neural network, logistic regression, and threshold models.	Eng, J.	2002	12239027	Venous thromboembolism
Neural networks as a prognostic tool of surgical risk in lung resections.	Esteva, H.	2002	12022553	Surgical complications
Role of ventilation scintigraphy in diagnosis of acute pulmonary embolism: an evaluation using artificial neural networks.	Evander, E.	2003	12748832	Diagnostic error; Venous thromboembolism
Enhancing the fever workup utilizing a multi-technique modeling approach to diagnose infections more accurately.	Fadlalla, A. M. A.	2012	20666579	Healthcare-associated infections
Predicting risk for portal vein thrombosis in acute pancreatitis patients: A comparison of radical basis function artificial neural network and logistic regression models.	Fei, Y.	2017	28246056	Venous thromboembolism
Artificial neural networks predict the incidence of portosplenomesenteric venous thrombosis in patients with acute pancreatitis.	Fei, Y.	2017	27960048	Venous thromboembolism
Risk prediction for portal vein thrombosis in acute pancreatitis using radial basis function.	Fei, Y.	2018	28943487	Venous thromboembolism
Letter to the editor: predicting central line-associated bloodstream infections and mortality using supervised machine learning.	Ferdoash, A.	2018	29778397	Healthcare-associated infections
Lateral inhibition in accumulative computation and fuzzy sets for human fall pattern recognition in colour and infrared imagery.	Fernandez-Caballero, A.	2013	24294142	Falls
Risk assessment for venous thromboembolism in chemotherapy-treated ambulatory cancer patients.	Ferroni, P.	2017	27491558	Venous thromboembolism
Predicting asthma exacerbations using artificial intelligence.	Finkelstein, J.	2013	23823374	Decompensation
Summarizing drug information in Medline citations.	Fiszman, M.	2006	17238342	Adverse drug events
Predicting survival of patients with sepsis by use of regression and neural network models.	Flanagan, J. R.	1996	10156949	Decompensation
Deep-learning model for predicting 30-day postoperative mortality.	Fritz, B. A.	2019	31558311	Surgical complications
Development of intelligent model to determine favorable wheelchair tilt and recline angles for people with spinal cord injury.	Fu, J.	2011	22254738	Pressure ulcers
Towards an intelligent system for clinical guidance on wheelchair tilt and recline usage.	Fu, J.	2012	23366964	Pressure ulcers
Development of intelligent model for personalized guidance on wheelchair tilt and recline usage for people with spinal cord injury: methodology and preliminary report.	Fu, J.	2014	25333817	Pressure ulcers

Towards the consideration of diagnostic delay in model-based clinical decision support.	Gaebel, J.	2017	29295404	Diagnostic error
The use of natural language processing on pediatric diagnostic radiology reports in the electronic health record to identify deep venous thrombosis in children.	Galvez, J. A.	2017	28815363	Venous thromboembolism
Visual search in breast imaging.	Gandomkar, Z.	2019	31287719	Diagnostic error
A concept for graph-based temporal similarity of patient data.	Ganzinger, M.	2019	31437901	Decompensation
Model-based and model-free machine learning techniques for diagnostic prediction and classification of clinical outcomes in Parkinson's Disease.	Gao, C.	2018	29740058	Falls
Automatic detection of surgical haemorrhage using computer vision.	Garcia-Martinez, A.	2017	28764873	Surgical complications
Feasibility of a real-time hand hygiene notification machine learning system in outpatient clinics.	Geilleit, R.	2018	29649558	Healthcare-associated infections
Text mining electronic health records to identify hospital adverse events.	Gerdes, L. U.	2013	23920919	Falls; Healthcare-associated infections; Pressure ulcers; Surgical complications
Patient-specific early classification of multivariate observations.	Ghalwash, M. F.	2015	26336666	Decompensation; Diagnostic error
Design and evaluation of a smart medication recommendation system for the electronic prescription.	Ghasemi, S. H.	2019	31118328	Adverse drug events
Septic shock prediction for ICU patients via coupled HMM walking on sequential contrast patterns.	Ghosh, S.	2017	28011233	Decompensation
Assessment of fall-risk by means of a neural network based on parameters assessed by a wearable device during posturography.	Giansanti, D.	2008	17560825	Falls
Cervical false negative cases detected by neural network-based technology. Critical review of cytologic errors.	Giovagnoli, M. R.	2002	12462090	Diagnostic error
Cranial reconstruction after decompressive craniectomy: prediction of complications using fuzzy logic.	Godil, S. S.	2011	21772193	Surgical complications
Automated detection of harm in healthcare with information technology: a systematic review.	Govindan, M.	2010	20671081	Adverse drug events; Healthcare-associated infections; Pressure ulcers; Surgical complications
Prediction of postoperative opioid analgesia using clinical-experimental parameters and electroencephalography.	Gram, M.	2017	27470494	Surgical complications
Ontological approach for safe and effective polypharmacy prescription.	Grando, A.	2012	23304299	Adverse drug events
A multi-layer monitoring system for clinical management of congestive heart failure.	Guidi, G.	2015	26391638	Decompensation
From vital signs to clinical outcomes for patients with sepsis: a machine learning basis for a clinical decision support system.	Gultepe, E.	2014	23959843	Decompensation
Use of computerized surveillance to detect nosocomial pneumonia in neonatal intensive care unit patients.	Haas, J. P.	2005	16216656	Healthcare-associated infections
Predicting ventriculoperitoneal shunt infection in children with hydrocephalus using artificial neural network.	Habibi, Z.	2016	27638720	Surgical complications
Toward a two-tier clinical warning system for hospitalized patients.	Hackmann, G.	2011	22195105	Decompensation
A model for detecting balance impairment and estimating falls risk in the elderly.	Hahn, M. E.	2005	16078620	Falls
Robust breast cancer prediction system based on rough set theory at National Cancer Institute of Egypt.	Hamouda, S. K. M.	2018	29157458	Diagnostic error
Review of a large clinical series: Predicting death for patients with abdominal septic shock.	Hanisch, E.	2011	21262751	Decompensation
Cardiologist-level arrhythmia detection and classification in ambulatory electrocardiograms using a deep neural network.	Hannun, A. Y.	2019	30617320	Decompensation

Towards vision-based smart hospitals: a system for tracking and monitoring hand hygiene compliance.	Haque, A.	2017	Haque, A. et al. Towards vision-based smart hospitals: a system for tracking and monitoring hand hygiene compliance. *Mach. Learn. Healthc. Conf*. **68**, 1-13 (2017).	Healthcare-associated infections
Artificial intelligence in surgery: promises and perils.	Hashimoto, D. A.	2018	29389679	Surgical complications
Computer vision analysis of intraoperative video: Automated recognition of operative steps in laparoscopic sleeve.	Hashimoto, D. A.	2019	31274652	Surgical complications
Multivariate computational analysis of biosensor's data for improved CD64 quantification for sepsis diagnosis.	Hassan, U.	2018	29564463	Decompensation; Diagnostic error
Extracting drug-drug interaction from the biomedical literature using a stacked generalization-based approach.	He, L.	2013	23785452	Adverse drug events
Neural networks for prognostication of patients with heart failure.	Hearn, J.	2018	30354561	Decompensation
Artificial neural networks for recognition of electrocardiographic lead reversal.	Heden, B.	1995	7733003	Diagnostic error
In-bed posture classification using deep autoencoders.	Heydarzadeh, M.	2016	28269123	Pressure ulcers
A machine learning approach to predict early outcomes after pituitary adenoma surgery.	Hollon, T. C.	2018	30453460	Surgical complications
Diagnostic outcomes of esophageal cancer by artificial intelligence using convolutional neural networks.	Horie, Y.	2019	30120958	Diagnostic error
Creating an automated trigger for sepsis clinical decision support at emergency department triage using machine learning.	Horng, S.	2017	28384212	Decompensation
Formulation of a model for automating infection surveillance: algorithmic detection of central-line associated bloodstream infection.	Hota, B.	2010	20064800	Healthcare-associated infections
TADAA: towards automated detection of anaesthetic activity.	Houliston, B. R.	2011	21860884	Surgical complications
Wearable-sensor-based classification models of faller status in older adults.	Howcroft, J.	2016	27054878	Falls
Prospective fall-risk prediction models for older adults based on wearable sensors.	Howcroft, J.	2017	28358689	Falls
Analysis and comparison of sleeping posture classification methods using pressure sensitive bed system.	Hsia, C. C.	2009	19965072	Pressure ulcers
Body posture recognition and turning recording system for the care of bed bound patients.	Hsiao, R. S.	2015	26444814	Pressure ulcers
Sleeping posture recognition using fuzzy c-means algorithm.	Hsiao, R. S.	2018	30396347	Pressure ulcers
Intelligent postoperative morbidity prediction of heart disease using artificial intelligence techniques.	Hsieh, N. C.	2012	21184153	Surgical complications
Prediction of clinical deterioration in hospitalized adult patients with hematologic malignancies using a neural network model.	Hu, S. B.	2016	27532679	Decompensation
Predicting warfarin dosage from clinical data: a supervised learning approach.	Hu, Y. H.	2012	22537823	Adverse drug events
Decision tree-based learning to predict patient controlled analgesia consumption and readjustment.	Hu, Y. J.	2012	23148492	Adverse drug events
Post-operative bleeding risk stratification in cardiac pulmonary bypass patients using artificial neural network.	Huang, R. S. P.	2015	25887872	Surgical complications
Application of artificial neural networks in renal transplantation: classification of nephrotoxicity and acute cellular rejection episodes.	Hummel, A. D.	2010	20304167	Diagnostic error; Surgical complications
Artificial intelligence may help in predicting the need for additional surgery after endoscopic resection of T1 colorectal cancer.	Ichimasa, K.	2018	29272905	Diagnostic error
Challenges, issues and trends in fall detection systems.	Igual, R.	2013	23829390	Falls
Prediction of sepsis patients using machine learning approach: a meta-analysis.	Islam, M. M.	2019	30712598	Decompensation
Comparison between logistic regression and neural networks to predict death in patients with suspected sepsis in the emergency room.	Jaimes, F.	2005	15774048	Decompensation
Computer-assisted diagnosis system for breast cancer in computed tomography laser mammography (CTLM).	Jalalian, A.	2017	28429195	Diagnostic error
Advancing in-hospital clinical deterioration prediction models.	Jeffery, A. D.	2018	30173171	Decompensation
Challenges in using the Arden Syntax for computer-based nosocomial infection surveillance.	Jenders, RA.	2001	11825197	Healthcare-associated infections

Are mortality and acute morbidity in patients presenting with nonspecific complaints predictable using routine variables?.	Jenny, M. A.	2015	26375290	Diagnostic error
Heart rate variability analysis during central hypovolemia using wavelet transformation.	Ji, S. Y.	2013	23371800	Decompensation
Fall down detection under smart home system.	Juang, L. H.	2015	26276014	Falls
Identifying patients experiencing opioid-induced respiratory depression during recovery from anesthesia: the application of electronic monitoring devices.	Jungquist, C. R.	2019	31050151	Adverse drug events; Surgical complications
Falling in the elderly: Do statistical models matter for performance criteria of fall prediction? Results from two large population-based studies.	Kabeshova, A.	2016	26686927	Falls
Learning representations for the early detection of sepsis with deep neural networks.	Kam, H. J.	2017	28843829	Decompensation
applying artificial intelligence to identify physiomarkers predicting severe sepsis in the PICU.	Kamaleswaran, R.	2018	30052552	Decompensation
Automated identification of innocent still's murmur in children.	Kang, S.	2017	27576242	Diagnostic error
Prediction of valve-related complications for artificial heart valves using adaptive neural networks: a preliminary study.	Katz, AS.	1993	8269158	Surgical complications
Prognostics of surgical site infections using dynamic health data.	Ke, C.	2017	27825798	Surgical complications
Supervised machine-learning predictive analytics for prediction of postinduction hypotension.	Kendale, S.	2018	30074930	Surgical complications
Review of fall detection techniques: a data availability perspective.	Khan, S. S.	2017	27889391	Falls
Computerized surveillance for adverse drug events in a pediatric hospital.	Kilbridge, P. M.	2008	18998917	Adverse drug events
Computerized surveillance for adverse drug events in a pediatric hospital.	Kilbridge, P. M.	2009	19567791	Adverse drug events
Predicting surgical complications in patients undergoing elective adult spinal deformity procedures using machine learning.	Kim, J. S.	2018	30348356	Surgical complications
Examining the ability of artificial neural networks machine learning models to accurately predict complications following posterior lumbar spine fusion.	Kim, J. S.	2018	29016439	Surgical complications
New phenotypes for sepsis: the promise and problem of applying machine learning and artificial intelligence in clinical research.	Knaus, W. A.	2019	31104067	Decompensation
Advances in infection surveillance and clinical decision support with fuzzy sets and fuzzy logic.	Koller, W.	2015	26262058	Healthcare-associated infections
The artificial intelligence clinician learns optimal treatment strategies for sepsis in intensive care.	Komorowski, M.	2018	30349085	Decompensation
Machine learning models to predict disease progression among veterans with hepatitis C virus.	Konerman, M. A.	2019	30608929	Diagnostic error
Dynamic Bayesian networks for context-aware fall risk assessment.	Koshmak, G.	2014	24859032	Falls
A knowledge engineering framework towards clinical support for adverse drug event prevention: the PSIP approach.	Koutkias, V.	2009	19745243	Adverse drug events
Constructing clinical decision support systems for adverse drug event prevention: a knowledge-based approach.	Koutkias, V.	2010	21347009	Adverse drug events
Towards a light-weight query engine for accessing health sensor data in a fall prevention system.	Kreiner, K.	2014	25160350	Falls
Advanced integrated real-time clinical displays.	Kruger, GH.	2011	21871406	Decompensation
Natural language processing to identify foley catheter-days.	Kudesia, V.	2012	23143371	Healthcare-associated infections
Predicting hospital-acquired pneumonia among schizophrenic patients: a machine learning approach.	Kuo, K. M.	2019	30866913	Healthcare-associated infections
Metabolite profiling of Clostridium difficile ribotypes using small molecular weight volatile organic compounds.	Kuppusami, S.	2015	Kuppusami, S et al. Metabolite profiling of Clostridium difficile ribotypes using small molecular weight volatile organic compounds. *Metabolomics.* **11**, 251–260 (2015).	Surgical complications
Wearable sensors in medical education: supporting hand hygiene training with a forearm EMG.	Kutafina, E.	2015	25980884	Healthcare-associated infections

Detection of tripping gait patterns in the elderly using autoregressive features and support vector machines.	Lai, D. T. H.	2008	18433757	Falls
On the feasibility of learning to predict minimum toe clearance under different walking speeds.	Lai, D. T. H.	2010	21096655	Falls
Prediction of foot clearance parameters as a precursor to forecasting the risk of tripping and falling.	Lai, D. T. H.	2012	21035220	Falls
Artificial intelligence techniques for monitoring dangerous infections.	Lamma, E.	2006	16445259	Healthcare-associated infections
Development and validation of a diagnostic model for early differentiation of sepsis and non-infectious SIRS in critically ill children - a data-driven approach using machine-learning algorithms.	Lamping, F.	2018	29544449	Decompensation; Diagnostic error
Neural network assessment of perioperative cardiac risk in vascular surgery patients.	Lapuerta, P.	1998	9456211	Surgical complications
Detection of correct and incorrect measurements in real-time continuous glucose monitoring systems by applying a postprocessing support vector machine.	Leal, Y.	2013	23380841	Decompensation
Development and Validation of a Deep Neural Network Model for Prediction of Postoperative In-hospital Mortality.	Lee, C. K.	2018	29664888	Surgical complications
Estimation of body postures on bed using unconstrained ECG measurements.	Lee, H. J.	2013	24240716	Pressure ulcers
The robot will clean up now. Rival technologies sans data compete for a growing market.	Lee, J.	2015	25671888	Healthcare-associated infections
Artificial intelligence versus logistic regression statistical modelling to predict cardiac complications after noncardiac surgery.	Lette, J.	1994	7834935	Surgical complications
Development and validation of an endoscopic images-based deep learning model for detection with nasopharyngeal malignancies.	Li, C.	2018	30253801	Diagnostic error
Using nursing information and data mining to explore the factors that predict pressure injuries for patients at the end of life.	Li, H. S.	2019	30418245	Pressure ulcers
The use of fuzzy backpropagation neural networks for the early diagnosis of hypoxic ischemic encephalopathy in newborns.	Li, L.	2011	21811381	Diagnostic error
Mining FDA drug labels for medical conditions.	Li, Q.	2013	23617267	Adverse drug events
Machine methods applied to predict ventilator-associated pneumonia with pseudomonas aeruginosa infection via sensor array of electronic nose in intensive care unit.	Liao, Y. H.	2019	31003541	Healthcare-associated infections
Automatic identification of methotrexate-induced liver toxicity in patients with rheumatoid arthritis from the electronic medical record.	Lin, C.	2015	25344930	Adverse drug events
Classification of older adults with/without a fall history using machine learning methods.	Lin, Z.	2015	26737845	Falls
Deep neural network improves fracture detection by clinicians.	Lindsey, R.	2018	30348771	Diagnostic error
Preventable complications and deaths after emergency nontrauma surgery.	Linnebur, M.	2018	30268169	Surgical complications
Determining molecular predictors of adverse drug reactions with causality analysis based on structure learning.	Liu, M.	2014	24334612	Adverse drug events
Machine learning and sepsis: on the road to revolution.	Liu, V. X.	2017	29028697	Decompensation
Using machine learning on home health care assessments to predict fall risk	Lo, Y.	2019	31438011	Falls
Machine-learned selection of psychological questionnaire items relevant to the development of persistent pain after breast cancer surgery.	Lotsch, J.	2018	30336857	Surgical complications
Machine-learning-derived classifier predicts absence of persistent pain after breast cancer surgery with high accuracy.	Lotsch, J.	2018	29876695	Surgical complications
Deep learning for fall detection: three-dimensional cnn combined with lstm on video kinematic data.	Lu, N.	2019	29994460	Falls
Personalized modeling for real-time pressure ulcer prevention in sitting posture.	Luboz, V.	2018	28637592	Pressure ulcers
An artificial neural network estimation of gait balance control in the elderly using clinical evaluations.	Lugade, V.	2014	24836062	Falls
Presymptomatic prediction of sepsis in intensive care unit patients.	Lukaszewski, R. A.	2008	18480235	Decompensation
Photographic LVAD driveline wound infection recognition using deep learning.	Luneburg, N.	2019	31118337	Diagnostic error; Healthcare-associated infections; Surgical complications
Posture detection based on smart cushion for wheelchair users.	Ma, C.	2017	28353684	Pressure ulcers
Depth-based human fall detection via shape features and improved extreme learning machine.	Ma, X.	2014	25375688	Falls
Reducing false negatives in clinical practice: the role of neural network technology.	Mango, L. J.	1996	8885796	Diagnostic error
Medical decision support using machine learning for early detection of late-onset neonatal sepsis.	Mani, S.	2014	24043317	Decompensation
Multicentre validation of a sepsis prediction algorithm using only vital sign data in the emergency department, general ward and ICU.	Mao, Q.	2018	29374661	Decompensation
Decision support tools, systems, and artificial intelligence in cardiac imaging.	Massalha, S.	2018	29960612	Diagnostic error
The trail making test: a study of its ability to predict falls in the acute neurological in-patient population.	Mateen, B. A.	2018	29807453	Falls
Flexible, cluster-based analysis of the electronic medical record of sepsis with composite mixture models.	Mayhew, M. B.	2018	29196114	Decompensation

Real-time multidimensional temporal analysis of complex high volume physiological data streams in the neonatal intensive care unit.	McGregor, C.	2013	23920577	Decompensation; Diagnostic error
The use of expert systems for improving test use and enhancing the accuracy of diagnosis.	McNeely, M. D. D.	2002	12134475	Diagnostic error
Depth-based activity recognition in ICUs using convolutional and recurrent neural networks.	Mehra, R.	2017	Mehra, R., Bianconi, G. M., Yeung, S. & Fei-Fei, L. Depth-based activity recognition in ICUs using convolutional and recurrent neural networks. 1–9 http://cs231n.stanford.edu/reports/2017/pdfs/506.pdf.	Decompensation
Optimal intensive care outcome prediction over time using machine learning.	Meiring, C.	2018	30427913	Decompensation
Cloud-based smart health monitoring system for automatic cardiovascular and fall risk assessment in hypertensive patients.	Melillo, P.	2015	26276015	Falls
Machine learning for real-time prediction of complications in critical care: a retrospective study.	Meyer, A.	2018	30274956	Decompensation
Fall detection using smart floor sensor and supervised learning.	Minvielle, L.	2017	29060638	Falls
Development and validation of a predictive model to identify individuals likely to have undiagnosed chronic obstructive pulmonary disease using an administrative claims database.	Moretz, C.	2015	26679964	Diagnostic error
Prediction of adverse events in patients undergoing major cardiovascular procedures.	Mortazavi, B. J.	2017	28287993	Healthcare-associated infections; Surgical complications
Running on empty? The compensatory reserve index.	Moulton, S. L.	2013	24256681	Decompensation
Reconstruction of the genomes of drug-resistant pathogens for outbreak investigation through metagenomic sequencing.	Mu, A.	2019	30651402	Healthcare-associated infections
Can we make a carpet smart enough to detect falls?	Muheidat, F.	2016	28269470	Falls
Identifying diagnostic errors with induced decision trees.	Murphy, C. K.	2001	11575486	Diagnostic error
Machine learning to predict venous thrombosis in acutely ill medical patients.	Nafee, T.	2020	32110753	Venous thromboembolism
Deep learning to predict falls in older adults based on daily-life trunk accelerometry.	Nait, A.	2018	29786659	Falls

A deep learning system for automatically identifying critical view of safety in laparoscopic cholecystectomy videos for assessment.	Namazi, B.	2017	Namazi, B., Sankaranarayanan, G., Devarajan, V. & Fleshman, J. A deep learning system for automatically identifying critical view of safety in laparoscopic cholecystectomy videos for assessment. In SAGES 2017 Annual Meeting (2017).	Surgical complications
A knowledge-based platform for assessing potential adverse drug reactions at the point of care: user requirements and design.	Natsiavas, P.	2019	31438076	Adverse drug events
An interpretable machine learning model for accurate prediction of sepsis in the ICU.	Nemati, S.	2018	29286945	Decompensation
E-pharmacovigilance: development and implementation of a computable knowledge base to identify adverse drug reactions.	Neubert, A.	2013	23586589	Adverse drug events
Effects of plasma transfusion on perioperative bleeding complications: a machine learning approach.	Ngufor, C.	2015	26262146	Surgical complications
A decision support system for pathology test result reviews in an emergency department to support patient safety and increase efficiency.	Nguyen, A.	2019	31438020	Diagnostic error
Prediction of postoperative pulmonary complications.	Nijbroek, S. G.	2019	30893115	Surgical complications
Towards precision in HF pharmacotherapy.	Norgard, N. B.	2017	28190189	Adverse drug events
Semiautomated system for nonurgent, clinically significant pathology results.	O'Connor, S. D.	2018	29874687	Diagnostic error
Towards a clinical decision support system for drug allergy management: are existing drug reference terminologies sufficient for identifying substitutes and cross-reactants?	Ogallo, W.	2015	26262387	Adverse drug events
Classification of nervous system withdrawn and approved drugs with ToxPrint features via machine learning strategies.	Onay, A.	2017	28325450	Adverse drug events
Addressing the flaws of current critical alarms: a fuzzy constraint satisfaction approach.	Otero, A.	2009	19796924	Diagnostic error
Machine learning to predict, detect, and intervene older adults vulnerable for adverse drug events in the emergency department.	Ouchi, K.	2018	29858745	Adverse drug events
Detecting falls with wearable sensors using machine learning techniques.	Ozdemir, AT.	2014	24945676	Falls
Evaluation of a model for glycemic prediction in critically ill surgical patients.	Pappada, S. M.	2013	23894489	Decompensation
Supervised machine learning in critical care: the path forward.	Parreco, J. P.	2018	29804636	Healthcare-associated infections
Predicting central line-associated bloodstream infections and mortality using supervised machine learning.	Parreco, J. P.	2018	29486341	Healthcare-associated infections
Neural network analysis of the volumetric capnogram to detect pulmonary embolism.	Patel, M. M.	1999	10559095	Venous thromboembolism
Application of an artificial intelligence program to therapy of high-risk surgical patients.	Patil, R. S.	1996	8968986	Surgical complications
Neural network in the clinical diagnosis of acute pulmonary embolism.	Patil, S.	1993	8252942	Diagnostic error; Venous thromboembolism
Prediction of specific pathogens in patients with sepsis: evaluation of TREAT, a computerized decision support system.	Paul, M.	2007	17449883	Decompensation
Artificial neural network-based pharmacogenomic algorithm for warfarin dose optimization.	Pavani, A.	2016	26666467	Adverse drug events
Developing predictive models using electronic medical records: challenges and pitfalls.	Paxton, C.	2013	24551396	Decompensation
Using artificial neural networks to predict potential complications during trauma patients' hospitalization period.	Pearl, A.	2009	19745384	Decompensation; Healthcare-associated infections
Decision support in trauma management: predicting potential cases of ventilator associated pneumonia.	Pearl, A.	2012	22874201	Diagnostic error; Healthcare-associated infections

Vision-based absence seizure detection.	Pediaditis, M.	2012	23365833	Diagnostic error
Predicting postoperative nausea and vomiting with the application of an artificial neural network.	Peng, S. Y.	2007	17065170	Surgical complications
A review of recent advances in data analytics for post-operative patient deterioration detection.	Petit, C.	2018	28828569	Decompensation; Surgical complications
Predictors of post-operative cardiovascular events, focused on atrial fibrillation, after valve surgery for primary mitral regurgitation.	Pimor, A.	2019	29608669	Surgical complications
Context-sensitive autoassociative memories as expert systems in medical diagnosis.	Pomi, A.	2006	17121675	Decompensation; Diagnostic error
An event-triggered machine learning approach for accelerometer-based fall detection.	Putra, I. P. E. S.	2017	29271895	Falls
Adverse drug event monitoring with clinical and laboratory data using arden syntax.	Rappelsberger, A.	2017	29295277	Adverse drug events
Elderly fall risk prediction based on a physiological profile approach using artificial neural networks.	Razmara, J.	2018	28050920	Falls
Simplified risk score models accurately predict the risk of major in-hospital complications following percutaneous coronary intervention.	Resnic, F. S.	2001	11423050	Surgical complications
Sepsis mortality prediction with the quotient basis kernel.	Ribas, R.	2014	24726036	Decompensation
Severe sepsis mortality prediction with relevance vector machines.	Ribas, V. J.	2011	22254260	Decompensation
Computerized detection of nosocomial infections in newborns.	Rocha, B. H.	1994	7950013	Healthcare-associated infections
Personalized and automated remote monitoring of atrial fibrillation.	Rosier, A.	2016	26487670	Decompensation
Neural hypernetwork approach for pulmonary embolism diagnosis.	Rucco, M.	2015	26515513	Venous thromboembolism
Palivizumab prophylaxis during nosocomial outbreaks of respiratory syncytial virus in a neonatal intensive care unit: predicting effectiveness with an artificial neural network model.	Saadah, L. M.	2014	23897635	Healthcare-associated infections
Evaluation of comfort associated with the use of a robotic mattress with an interface pressure mapping system and automatic inner air-cell pressure adjustment function in healthy volunteers.	Saegusa, M.	2018	29910093	Pressure ulcers
Development of an early diagnostic system using fuzzy theory for postoperative infections in patients with gastric cancer.	Sakaguchi, S.	2004	15237253	Healthcare-associated infections; Surgical complications
Validation of an online risk calculator for the prediction of anastomotic leak after colon cancer surgery and preliminary exploration of artificial intelligence-based analytics.	Sammour, T.	2017	29080956	Surgical complications
Comparison of variable selection methods for clinical predictive modeling.	Sanchez-Pinto, L. N.	2018	29887230	Decompensation
A prognostic model of surgical site infection using daily clinical wound assessment.	Sanger, P. C.	2016	27188832	Surgical complications
Computing network-based features from physiological time series: application to sepsis detection.	Santaniello, S.	2014	25570825	Decompensation
A machine learning approach to estimate minimum toe clearance using inertial measurement units	Santhiranayagam, B. K.	2015	26573902	Falls
Prediction of postoperative morbidity after lung resection using an artificial neural network ensemble.	Santos-Garcia, G.	2004	14684265	Surgical complications
Individualized sepsis treatment using reinforcement learning.	Saria, S.	2018	30397359	Decompensation
Electronic nose in the detection of wound infection bacteria from bacterial cultures: a proof-of-principle study.	Saviauk, T.	2018	29320769	Surgical complications
Computer-assisted decision support for the diagnosis and treatment of infectious diseases in intensive care units.	Schurink, C. A. M.	2005	15854886	Healthcare-associated infections
The promise and perils of wearable physiological sensors for diabetes management.	Schwartz, F.	2018	29542348	Decompensation
Classification of patients with sepsis according to blood genomic endotype: a prospective cohort study.	Scicluna, B. P.	2017	28864056	Decompensation
Machine learning for predicting sepsis in-hospital mortality: an important start.	Scott, H.	2016	27163688	Decompensation
Neural network analysis of ventilation-perfusion lung scans.	Scott, J. A.	1993	8430170	Venous thromboembolism
Using artificial neural network analysis of global ventilation-perfusion scan morphometry as a diagnostic tool.	Scott, J. A.	1999	10511154	Diagnostic error; Venous thromboembolism
How well can radiologists using neural network software diagnose pulmonary embolism?.	Scott, J. A.	2000	10915682	Diagnostic error; Venous thromboembolism
Pulmonary perfusion patterns and pulmonary arterial pressure.	Scott, J. A.	2002	12147850	Diagnostic error; Venous thromboembolism

Artificial intelligence: its use in medical diagnosis.	Scott, R.	1993	8441047	Diagnostic error; Venous thromboembolism
Adopting a smart toothbrush with artificial intelligence may improve oral care in patients admitted to the intensive care unit.	Scquizzato, T.	2019	31215441	Healthcare-associated infections
How doctors think, and how software can help avoid cognitive errors in diagnosis.	Segal, M.	2007	17850393	Diagnostic error
Automated lung outline reconstruction in ventilation-perfusion scans using principal component analysis techniques.	Serpen, G.	2003	12565727	Venous thromboembolism
A knowledge-based artificial neural network classifier for pulmonary embolism diagnosis.	Serpen, G.	2008	18022148	Venous thromboembolism
Fuzzy logic in neurosurgery: predicting poor outcomes after lumbar disk surgery in 501 consecutive patients.	Shamim, M. S.	2009	20082825	Surgical complications
Java-based diabetes type 2 prediction tool for better diagnosis.	Shankaracharya, O.	2012	22059431	Diagnostic error
Artificial neural networking model for the prediction of early occlusion of bilateral plastic stent placement for inoperable hilar cholangiocarcinoma.	Shao, F.	2018	29252936	Surgical complications
An integrated framework for identification of effective and synergistic anti-cancer drug combinations.	Sharma, A.	2018	30304987	Adverse drug events
Aiding the digital mammogram for detecting the breast cancer using shearlet transform and neural network.	Shenbagavalli, P.	2018	30256567	Diagnostic error
Hemodynamic and oxygen transport patterns for outcome prediction, therapeutic goals, and clinical algorithms to improve outcome. Feasibility of artificial intelligence to customize algorithms.	Shoemaker, W. C.	1992	1424937	Surgical complications
Prediction of allogeneic hematopoietic stem-cell transplantation mortality 100 days after transplantation using a machine learning algorithm: a european group for blood and marrow transplantation acute leukemia working party retrospective data mining study	Shouval, R.	2015	26240227	Surgical complications
Time-domain heart rate variability-based computer-aided prognosis of lung cancer.	Shukla, R. S.	2018	30147095	Diagnostic error
Emerging technologies for molecular diagnosis of sepsis.	Sinha, M.	2018	29490932	Decompensation
Predicting neurosurgical outcomes in focal epilepsy patients using computational modelling.	Sinha, N.	2017	28011454	Surgical complications
Automated surveillance of healthcare-associated infections: state of the art.	Sips, M. E.	2017	28505027	Healthcare-associated infections
Computer-aided, case-based diagnosis of mammographic regions of interest containing microcalcifications.	Sklansky, J.	2000	10845398	Diagnostic error
Evaluation of a novel system to enhance clinicians' recognition of preadmission adverse drug reactions.	Smith, J. C.	2018	29742757	Adverse drug events
Data-driven temporal prediction of surgical site infection.	Soguero-Ruiz, C.	2015	26958256	Surgical complications
Support vector feature selection for early detection of anastomosis leakage from bag-of-words in electronic health records.	Soguero-Ruiz, C.	2016	25312965	Surgical complications
Predicting colorectal surgical complications using heterogeneous clinical data and kernel methods.	Soguero-Ruiz, C.	2016	26980235	Surgical complications
Detection of clinically important colorectal surgical site infection using Bayesian network.	Sohn, S.	2017	28032554	Healthcare-associated infections; Surgical complications
Predicting developmental disorder in infants using an artificial neural network.	Soleimani, F.	2013	23852837	Diagnostic error
Reducing the toxicity risk in antibiotic prescriptions by combining Oontologies with a multiple criteria decision model.	Souissi, S. B.	2017	29854233	Adverse drug events
Feasibility of applying data mining techniques for predicting technical difficulties during laparoscopic cholecystectomy based on routine patient work-up in a small community hospital.	Stanisic, V.	2013	24052489	Surgical complications
Compensatory reserve for early and accurate prediction of hemodynamic compromise: case studies for clinical utility in acute care and physical performance.	Stewart, C.	2016	27045488	Decompensation
Data mining techniques for assisting the diagnosis of pressure ulcer development in surgical patients.	Su, C. T.	2012	21503743	Pressure ulcers
Clinical intervention prediction and understanding with deep neural networks.	Suresh, H.	2017	Suresh, H. et al. Clinical intervention prediction and understanding using deep networks. *Mach. Learn. Healthc. Conf* . **68,** 1–16 (2017).	Decompensation

Development and validation of a novel molecular biomarker diagnostic test for the early detection of sepsis.	Sutherland, A.	2011	21682927	Decompensation
Artificial neural networks in nuclear medicine.	Swietlik, D.	2004	15318313	Diagnostic error
A self-administered screening instrument for psychogenic nonepileptic seizures.	Syed, T. U.	2009	19433737	Diagnostic error
Comparison of 2 natural language processing methods for identification of bleeding among critically ill patients.	Taggart, M.	2018	30646240	Decompensation
Autoregressive-moving-average hidden Markov model for vision-based fall prediction-An application for walker robot.	Taghvaei, S.	2017	27450279	Falls
Improving end of life care: an information systems approach to reducing medical errors.	Tamang, S.	2005	15923765	Diagnostic error
Combining biomarkers with EMR data to identify patients in different phases of sepsis.	Taneja, I.	2017	28883645	Decompensation
Prediction of in-hospital mortality in emergency department patients with sepsis: a local big data-driven, machine learning approach.	Taylor, R. A.	2016	26679719	Decompensation
Predicting urinary tract infections in the emergency department with machine learning.	Taylor, R. A.	2018	29513742	Diagnostic error
Application of machine learning techniques to high-dimensional clinical data to forecast postoperative complications.	Thottakkara, P.	2016	27232332	Decompensation; Surgical complications
Automated extraction of vte events from narrative radiology reports in electronic health records: a validation study.	Tian, Z.	2017	25924079	Venous thromboembolism
Use of machine learning theory to predict the need for femoral nerve block following ACL repair.	Tighe, P.	2011	21899717	Surgical complications
Use of machine-learning classifiers to predict requests for preoperative acute pain service consultation.	Tighe, P.	2012	22958457	Surgical complications
Teaching a machine to feel postoperative pain: combining high-dimensional clinical data with machine learning algorithms to forecast acute postoperative pain.	Tighe, P. J.	2015	26031220	Surgical complications
Detection of lung cancer in exhaled breath with an electronic nose using support vector machine analysis.	Tirzite, M.	2017	28585921	Diagnostic error
Predictive analytics for identification of patients at risk for QT interval prolongation: a systematic review.	Tomaselli, M.	2018	29882591	Decompensation
Acute pulmonary embolism: artificial neural network approach for diagnosis.	Tourassi, G. D.	1993	8210389	Venous thromboembolism
The effect of data sampling on the performance evaluation of artificial neural networks in medical diagnosis.	Tourassi, G. D.	1997	9107614	Diagnostic error; Venous thromboembolism
Acute pulmonary embolism: cost-effectiveness analysis of the effect of artificial neural networks on patient care.	Tourassi, G. D.	1998	9423655	Venous thromboembolism
Multifractal texture analysis of perfusion lung scans as a potential diagnostic tool for acute pulmonary embolism.	Tourassi, G. D.	2001	11058691	Diagnostic error; Venous thromboembolism
Eigen posture based fall risk assessment system using kinect.	Tripathy, SR.	2018	30440310	Falls
Adverse outcome in surgery for chronic leg ischaemia--risk factors and risk prediction when using different statistical methods.	Troeng, T.	1992	1451819	Surgical complications
Morphological and wavelet features towards sonographic thyroid nodules evaluation.	Tsantis, S.	2009	19111442	Diagnostic error
On the wearable sensor data fusion to a single sensor machine learning technique in fall detection.	Tsinganos, P.	2018	29443923	Falls
Machine learning classification of medication adherence in patients with movement disorders using non-wearable sensors.	Tucker, C.	2015	26406881	Adverse drug events
Learning approaches to improve prediction of drug sensitivity in breast cancer patients.	Turki, T.	2016	28269014	Adverse drug events
Abciximab pharmacodynamic model with neural networks used to integrate sources of patient variability.	Urquidi-Macdonald, M.	2004	14749692	Adverse drug events
A minimal set of physiomarkers in continuous high frequency data streams predict adult sepsis onset earlier.	van Wyk, F.	2019	30623784	Decompensation
Quantifying surgical complexity with machine learning: looking beyond patient factors to improve surgical models.	Van, E.	2014	25108343	Healthcare-associated infections; Surgical complications
Relevance of deep learning to facilitate the diagnosis of HER2 status in breast cancer.	Vandenberghe, M. E.	2017	28378829	Diagnostic error
A framework for patient state tracking by classifying multiscalar physiologic waveform features.	Vandendriessche, B.	2017	28328498	Decompensation
Machine learning in critical care: state-of-the-art and a sepsis case study.	Vellido, A.	2018	30458795	Decompensation
Improved diagnosis of breast implant rupture with sonographic findings and artificial neural networks.	Venta, L. A.	1998	9561256	Diagnostic error
Prognostic Bayesian networks II: an application in the domain of cardiac surgery.	Verduijn, M.	2007	17709302	Surgical complications
Binary tissue classification on wound images with neural networks and bayesian classifiers.	Veredas, F.	2010	19825516	Pressure ulcers
Processing gradual information with fuzzy arden syntax.	Vetterlein, T.	2010	20841802	Healthcare-associated infections
Identifying predictive features in drug response using machine learning: opportunities and challenges.	Vidyasagar, M.	2015	25423479	Adverse drug events
Predicting macular edema recurrence from spatio-temporal signatures in optical coherence tomography images.	Vogl, W. D.	2017	28475051	Diagnostic error

Predicting nocturnal hypoglycemia from continuous glucose monitoring data with extended prediction horizon.	Vu, L.	2019	32308884	Adverse drug events
Prediction of severe sepsis using SVM model.	Wang, S. L.	2010	20865488	Decompensation
Automatic learning of mortality in a CPN model of the systemic inflammatory response syndrome.	Ward, L.	2017	27833000	Decompensation
Classification of hospital acquired complications using temporal clinical information from a large electronic health record.	Warner, J. L.	2016	26707449	Surgical complications
Usefulness of a computerized expert system associated with systematic O-serotyping for the early detection of outbreaks of hospital acquired infections and for the presumptive antibiotic therapy of Pseudomonas aeruginosa infections.	Watine, J.	1996	8761597	Healthcare-associated infections
Improving diagnostic accuracy using a hierarchical neural network to model decision subtasks.	West, D.	2000	10708254	Diagnostic error
Intelligent alarms reduce anesthesiologist's response time to critical faults.	Westenskow, D. R.	1992	1466459	Diagnostic error
Artificial intelligence: an inkling of caution.	Wetzel, R. C.	2018	30281572	Decompensation
A web-based system for clinical decision support and knowledge maintenance for deterioration monitoring of hemato-oncological patients.	Wicht, A.	2013	23522434	Decompensation; Diagnostic error; Healthcare-associated infections
The use of artificial neural network analysis can improve the risk-stratification of patients presenting with suspected deep vein thrombosis.	Willan, J.	2019	Willan, J., Katz, H. & Keeling, D. The use of artificial neural network analysis can improve the risk-stratification of patients presenting with suspected deep vein thrombosis. *Br. J. Haematol*. **185**, 289–296 (2019).	Venous thromboembolism
Prediction of prolonged ventilation after coronary artery bypass grafting: data from an artificial neural network.	Wise, E. S.	2017	28263144	Surgical complications
Computer-assisted detection of pulmonary embolism: evaluation of pulmonary CT angiograms performed in an on-call setting.	Wittenberg, R.	2010	19862534	Venous thromboembolism
Natural language processing and its implications for the future of medication safety: a narrative review of recent advances and challenges.	Wong, A.	2018	29884988	Adverse drug events
Predicting postoperative vomiting among orthopedic patients receiving patient-controlled epidural analgesia using SVM and LR.	Wu, H. Y.	2016	27247165	Surgical complications
Combining support vector machine with genetic algorithm to classify ultrasound breast tumor images.	Wu, W. J.	2012	22939834	Diagnostic error
Adverse drug event detection from electronic health records using hierarchical recurrent neural networks with dual-level embedding.	Wunnava, S.	2019	30649736	Adverse drug events
Evaluation of feature extraction and recognition for activity monitoring and fall detection based on wearable EMG sensors.	Xi, X.	2017	28555016	Falls
DINIES: drug-target interaction network inference engine based on supervised analysis.	Yamanishi, Y.	2014	24838565	Adverse drug events
Use of an artificial neural network to differentiate between ECGs with IRBBB patterns of atrial septal defect and healthy subjects.	Yang, S.	2002	12509123	Diagnostic error
Leveraging reporting through knowledge support: a knowledge-based approach to promoting patient fall prevention.	Yao, B.	2017	29295245	Falls
Bedside computer vision - moving artificial intelligence from driver assistance to patient safety.	Yeung, S.	2018	29617592	Falls; Healthcare-associated infections; Surgical complications
Establishing a classification system for high fall-risk among inpatients using support vector machines.	Yokota, S.	2017	28800580	Falls
Jump neural network for real-time prediction of glucose concentration.	Zecchin, C.	2015	25502386	Decompensation
Developing and evaluating a machine learning based algorithm to predict the need of pediatric intensive care unit transfer for newly hospitalized children.	Zhai, H.	2014	24813568	Decompensation
Gastric precancerous diseases classification using CNN with a concise model.	Zhang, X.	2017	28950010	Diagnostic error
Classification of whole mammogram and tomosynthesis images using deep convolutional neural networks.	Zhang, X.	2018	29994219	Diagnostic error

A single kernel-based approach to extract drug-drug interactions from biomedical literature.	Zhang, Y.	2012	23133662	Adverse drug events
Handling temporality of clinical events for drug safety surveillance.	Zhao, J.	2015	26958278	Adverse drug events
A graph kernel based on context vectors for extracting drug-drug interactions.	Zheng, W.	2016	27012903	Adverse drug events
An attention-based effective neural model for drug-drug interactions extraction.	Zheng, W.	2017	29017459	Adverse drug events
Predicting adverse drug reactions of combined medication from heterogeneous pharmacologic databases.	Zheng, Y.	2018	30598065	Adverse drug events
Classification analyses for prostate cancer, benign prostate hyperplasia and healthy subjects by SERS-based immunoassay of multiple tumour markers.	Zhou, L.	2018	30029370	Diagnostic error
Use of artificial neural network to predict warfarin individualized dosage regime in Chinese patients receiving low-intensity anticoagulation after heart valve replacement.	Zhou, Q.	2014	25164187	Surgical complications
Lung cancer cell identification based on artificial neural network ensembles.	Zhou, Z. H.	2002	11779683	Diagnostic error
Modeling polypharmacy side effects with graph convolutional networks.	Zitnik, M.	2018	29949996	Adverse drug events
